# Transcriptome remodelling and changes in growth and cardiometabolic phenotype result following *Grb10a* knockdown in the early life of the zebrafish

**DOI:** 10.1007/s00018-025-05784-9

**Published:** 2025-07-19

**Authors:** Bridget L. Evans, Terence Garner, Chiara De Leonibus, Lily Wright, Megan Sharps, Oliver H. Wearing, Daniel M. Ripley, Holly A. Shiels, Adam F. L. Hurlstone, Peter E. Clayton, Adam Stevens

**Affiliations:** 1 Division of Developmental Biology and Medicine, School of Medical Sciences, Faculty of Biology, Medicine, and Health, University of Manchester and the Manchester Academic Health Science Centre, Manchester, UK; 2 Division of Cardiovascular Sciences, School of Medical Sciences, Faculty of Biology Medicine and Health, University of Manchester, Manchester, UK; 3 Division of Infection, Immunity, and Respiratory Medicine, School of Biological Sciences, Faculty of Biology, Medicine, and Health, University of Manchester, Manchester, UK

**Keywords:** Transcriptome, Growth, Cardiometabolic, Development

## Abstract

Embryonic growth trajectory is a risk factor for chronic metabolic and cardiovascular disorders. Grb10 is a negative regulator of the main pathways driving embryonic growth. This study has characterised growth, cardiometabolic status, and the impact on co-ordination of gene expression following morpholino-induced embryonic and early larval knockdown (KD) of *grb10a* expression in zebrafish (*Danio rerio*). Grb10 knockdown was associated with increased embryonic growth and metabolic rate, and decreased heart rate in early life. Juvenile growth rate was also elevated. The transcriptome was assessed over 5 to 30 days post fertilisation, coinciding with major changes in zebrafish (ZF) maturation and development. Significant and persistent organisational and functional changes in the whole transcriptome over this time were evident, including dysregulation of multiple growth, cardiac, and metabolic pathways. In adulthood (18 months), KD ZF had greater body length and mass than controls, with elevated cardiac muscle content, an increased aerobic scope, and higher fasting glucose levels. This study demonstrates that early life disruption of a single gene in zebrafish can result in long-term transcriptomic remodelling and alterations to the adult cardiometabolic phenotype.

**Supplementary Information:**

The online version contains supplementary material available at 10.1007/s00018-025-05784-9.

## Introduction

The Developmental Origins of Health and Disease (DOHaD) hypothesis proposes that diseases occurring in adulthood have their origins during development [1–3]. It is now accepted that in humans a wide range of diseases have early developmental origins. Therefore, understanding the impact of an altered embryonic growth trajectory in an animal model is important for establishing the mechanisms that underpin DOHaD.

During embryonic development, temperature, circulating glucose levels, and oxygen availability serve as indicators of the external environment [2, 4, 5]. Small changes to the phenotype occur in response to external cues to promote immediate survival in a process termed “developmental plasticity”. While these correlations have been observed across many species, targeted, longitudinal *in-vivo* studies to elucidate DOHaD, and the pathways involved, are lacking.

Zebrafish are an ideal model organism for developmental study owing to a rapid generation time, large clutch sizes, and ease of access to embryos. Under normoxic conditions and at temperatures of ~ 28 °C, zebrafish embryogenesis occurs over three days and can be monitored in real time due to the transparent nature of the embryos [6]. The zebrafish progresses from embryo to adulthood (from sexual maturity onwards) within three months [7]. The larval stage ends following a period of rapid growth and physiological changes, termed “metamorphosis”. These life history stage transitions and gradual ageing process have parallels to those seen in humans [8]. Zebrafish have successfully been used to study the impact of early vitamin D deficiency on later life health [9], and diseases associated with ageing [8, 10, 11]. As in mammals, embryonic growth in zebrafish is primarily driven by the insulin/insulin like growth factor (Ins/IGF) signaling pathway [12].

Genomic approaches to DOHaD have focussed on epigenetic marks, and their correlation with early life exposures and later health outcomes [13]. Recently it has been shown that transcriptional organisation during fish embryogenesis is related to the developmental environment (elevated water temperature) and correlates with future gene expression changes [13]. This work suggests that a reorganisation of the whole transcriptome is generated by exposure to different, potentially transient, early life environments.

Growth factor receptor bound protein 10 (GRB10) is a negative regulator of the Insulin/IGF signaling pathway. GRB10 downregulates the growth response, promoting a switch from glucose to fat metabolism and halting cell cycle progression [14–16]. GRB10 expression limits placental growth and efficiency [17] and correlates with small body size [18] in mammals. In humans, genome-wide association studies (GWAS) show *GRB10* is associated with type 2 diabetes [19], and *GRB10* copy number variation is associated with Silver Russell Syndrome [20], a rare growth disorder typically characterised by intrauterine growth restriction, low birth weight for gestational age, hypoglycemia, and poor muscle development in childhood, with an increased risk of cardiometabolic disorders in adulthood. The role of GRB10 in the regulation of human growth is notably associated with response to recombinant human growth hormone in children with growth hormone deficiency, where lower GRB10 expression correlates with a greater response [13, 21].

Variability in *GRB10* expression is also linked to the dramatic range of body sizes observed between cetaceans [22]. Average daily mass gain is elevated in beef cattle with a *GRB10* associated deletion [23], and *GRB10* single nucleotide polymorphisms (SNPs) impact muscle and lipid mass and body conditioning score [24]. Global *Grb10* knockout in mice correlates with a “leaner” phenotype, including elevated muscle, reduced lipid mass, and insulin sensitivity [25, 26]. *GRB10* is therefore a relevant candidate gene to manipulate in early life to explore mechanisms that may associate with phenotypic changes consistent with the DOHaD hypothesis.

In this study, *grb10a* expression was suppressed in the embryonic and early larval stages of wild-type zebrafish by antisense oligonucleotide directed blocking of mRNA splicing. The importance of *grb10a* in the control of growth, metabolism, and cardiac health was assessed over the first 5 days post fertilisation (dpf). The impact of early life *grb10a* disruption on the organisation of the transcriptome was investigated over 5–30 days post fertilisation [dpf]. This analysis was performed by quantifying so called higher order interactions within the transcriptome where the relationships between more than two groups of genes are described using hypergraphs (standard network analysis examines interactions between pairs of genes). These hypergraphs are then used to characterise system dynamics, in this case transcriptomic coordination [13, 27–29]. Finally, body morphology, cardiac phenotype, and metabolism were assessed in adult life at 18 months of age.

## Materials and methods

### Zebrafish husbandry

AB zebrafish were maintained under standard conditions (≈28 °C; 14/10 h light/dark cycle; < 5 fish per litre) within the Biological Services Unit of The University of Manchester. Regulated procedures received institutional ethical approval and were performed under a Home Office Licence (PPL P005EFE9F9). To generate embryos, breeding pairs of similar ages were selected at a ratio of 1 male to 1 female and fasted overnight in breeding tanks. Dividers were removed at the start of the following light cycle and embryos were collected after 20 min of free breeding. Embryos were kept at a stocking density of < 50 per petri dish and raised in embryo water (Instant Ocean salt 60 µg/mL) up to 5 dpf and then transferred to the main aquarium. Zebrafish were fed as follows: from 5–14 dpf, an early morning feed of ZM000 (ZM Fish Food), mid-morning and midday feed of rotifers, and afternoon feed of ZM000; from 15–24 dpf, an early morning feed of ZM100, mid-morning and midday feed of a rotifers-brine shrimp cocktail, and afternoon feed of ZM100. From 25–59 dpf an early-morning feed of ZM100, mid-morning and midday feed of brine shrimp, and afternoon feed of ZM100. From 60 dpf, an early morning feed of ZM300, mid-morning and midday feed of brine shrimp, afternoon feed of ZM300.

### Knockdown of *grb10a* expression

Morpholino-modified antisense oligonucleotide knockdown (KD) of *grb10a* was validated in accordance with current guidelines for morpholino use in zebrafish [30]. Morpholinos targeting exon three (e3i3) and four (e4i4) were designed by and obtained from Gene Tools, LLC (Philomath, OR, USA) along with a standard control (SC) oligonucleotide targeting human β-globin, used to control for microinjection (sequences & microinjection solutions, Supplementary Table 1 [30]). Phenol red and nCerulean (nuclear-targeting blue fluorescent protein) mRNA were included to ensure successful injection. Embryos received a single injection into the yolk directly below the cell mass at the single-cell stage, as per established methods. Embryos were screened for fluorescence at 48 h post-fertilisation (hpf) to ensure constitutive and even uptake of the injection material. Non-uniformly or weakly-fluorescent embryos were removed. The use of two morpholinos with non-overlapping target sites establishes that the observed phenotype is replicable [31]. The strength of effect varies between morpholinos due to the affinity of the target site. Once validation was established, the morpholino with the greatest strength of effect (e3i3) was selected for longitudinal analysis.

### Validation of *grb10a* knockdown

*Grb10a* expression in relation to morpholino activity was examined over the first five days post-fertilisation, and *grb10a* exon expression in the transcriptomic data was examined over 5 to 30 days.

To confirm antisense oligonucleotide activity causing splicing interruption, primer sequences to introns 3 and 4, outlined in Supplementary Table 1, were designed using SnapGene® (GSL Biotech, San Diego, CA, USA) and synthesised by Thermo Fisher Scientific (Waltham, MA, USA). Specificity was confirmed using Primer BLAST [32]. RNA was extracted from pooled zebrafish embryos at 24, 48, 72, 96, and 120 hpf (*n* = 3, 5 embryos per pool). Extraction was performed using QIAGEN RNeasy lipid extraction kit according to the manufacturer’s instructions, and cDNA was generated by reverse transcription using the ProtoScript® II First Strand cDNA Synthesis Kit (NEB). cDNA was amplified with primers flanking each splice site by Taq polymerase (NEB, Hitchin, UK) (thermocycling parameters outlined in Supplementary Table 1). β-actin (*actb1*) was used as a positive control for cDNA integrity.

To assess *grb10a* expression over 5 to 30 days post fertilisation, analysis of the transcriptome was performed. Raw intensity values were background corrected, log2 normalised and quantile normalised before summarising intensities for all probes located in the *grb10a* gene, using the Robust Multi-Array (RMA) pipeline (*affy* package for* R* [33]). Probes annotated to *grb10a* were identified using the biomaRt package in R [34]. Expression of these probes was evaluated within each timepoint, using Wilcoxon rank sum tests to compare standard control (SC) with knockdown (KD.

### Confirming impact on downstream signalling by western blot

The effect of the morpholino activity on levels of phosphorylated AKT and S6, as markers of endogenous insulin signalling, was assessed in 96 hpf larvae. These larvae were deyolked in Ringer’s Buffer to limit background interference (*n* = 15, performed in triplicate pools), were resuspended in 100 μl RIPA buffer (150 mM NaCl, 1% Nonident P-40, 0.5% Sodium deoxycholate, 0.1% SDS, 25 mM Tris pH 7.4) containing protease and phosphatase inhibitors, and homogenised. Samples were incubated on ice for 30 min, clarified by centrifugation at 4 °C, and denatured at 98 °C for 5 min in Laemmli buffer (2% SDS, 10% glycerol, 60 mM Tris–Cl, 0.01% bromophenol blue, 0.1% β-Mercaptoethanol). Proteins were separated by 10% SDS acrylamide gel electrophoresis and transferred using established methods. The transfer membrane was incubated in blocking buffer (3% BSA in TBS-T) for one-hour, primary antibody (AKT and S6, Supplementary Table 1) at 4 °C under constant agitation overnight, and secondary antibody (Supplementary Table 1) for one hour at room temperature. The membrane was covered with ECL Western Blotting Substrate (Promega, Southampton, UK) and d immediately using a Bio-Rad Gel Doc Xr + imaging system. Protein expression was quantified from band intensity in ImageJ [35]. The signal is expressed as the proportion of active protein (phosphorylated) to total protein by dividing the intensity of the phosphorylated band by the intensity of the total band.

### *grb10a* mRNA for overexpression and rescue

Confirmation that grb10a mRNA injections caused embryonic growth reduction and that co-injection of e313 and *grb10a* mRNA reversed this was undertaken. Total RNA was extracted, and cDNA generated from a pool of 96 hpf embryos, as previously described. *Grb10a* was amplified from cDNA by high specificity PCR (NEB Q5 Hot Start) with primers flanking open reading frame (Supplementary Table 1). The product was purified using QIAGEN Quick Gel Extraction, blunt ligated into pCR-Blunt II-TOPO (Thermo Fisher Scientific, MA, USA) and transformed into *E. coli* following the NEB transformation protocol (New England Biolabs, MA, USA). The purified plasmid was Sanger sequenced to confirm successful cloning. The insert was liberated by digestion with *Cla I* and *Xba I* restriction enzymes (New England Biolabs, MA, USA) and subcloned into pCS2 + [36]. Capped RNA was generated using the mMESSAGE mMACHINE® SP6 Transcription Kit (Thermo Fisher Scientific, MA, USA) according to the manufacturer’s instructions. RNA was purified by MEGAclear™ Transcription Clean-Up (Thermo Fisher Scientific, MA, USA). 1 μl of the purified RNA was analysed by gel electrophoresis to confirm structural integrity. Injections were carried out as described above and embryonic length was measured at 48, 72, 96 and 120 hpf as described below. Lengths were expressed relative to standard controls.

### Embryonic physiological and metabolic measurements

Whole body length, the longest straight-line distance between the snout and tip of the notochord, and yolk area measurements were taken at 24-h intervals from 24 to 120 hpf for SC and e3i3 zebrafish and from 48 to 120 hpf for all other zebrafish. If chorionated, embryos were manually dechorionated with fine tipped forceps and acclimatised for ≥ one hour before imaging. Images were imported into ImageJ, which was calibrated with an image of a graticule of known size. Data were imported into GraphPad Prism version 7.00 for Windows (GraphPad Software, La Jolla, CA, USA, www.graphpad.com).

To investigate embryonic cardiac phenotype, embryos were sedated using an anaesthetic concentration known to have no impact on cardiac function (0.04% MS-222 solution [37]). Fish were imaged at room temperature, and heart beats were counted over a 20 s period and converted to beats per minute (bpm).

A Glucose Uptake-Glo TM Assay (Promega) was performed on 96 hpf zebrafish to detect differences in metabolic rate [38]. Individual embryos (*n* = 5 per treatment) were injected with 1 mM 2-deoxy-D-glucose directly into the yolk and allowed to recover for 30 min. Embryos were processed according to an established protocol [38]. Briefly, embryos were homogenised in stop solution and treated with detection reagent. Homogenates were incubated for 30 min at room temperature. Luminescence was measured using a Synergy™ H1 Microplate reader (BioTek Instruments, Inc., VT, USA, software version 2.07.17) with 8 readings per well. Readings were adjusted for background luminescence.

### Cell type deconvolution

In order to gain insight into the proportions of different tissue types in our transcriptomic data, Multi-subject Single Cell deconvolution (MuSiC) [39] was used, an approach which utilizes tissue or cell-type specific scRNA-seq data to estimate proportions in bulk sequencing data. ScRNA-seq data from WT zebrafish at 5dpf, annotated to the tissue level [40], was used to estimate the proportion of each tissue type in the 5–30 dpf bulk sequencing samples.

### Transcriptomic analysis

**Fig. 1:**
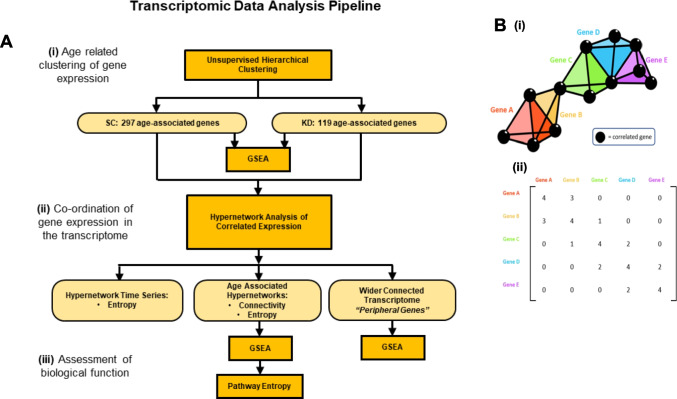
Pipeline of transcriptomic analysis and hypergraphs. (**A**) Analysis pipeline of transcriptomic data. **(i)** Unsupervised hierarchical clustering was performed to identify age-associated gene clusters. Genes were filtered by variance, using a projection score to maximise the informativeness of the genes selected. Clusters of age-associated genes were identified for SC and KD animals. **(ii)** hypergraphs were generated using age associated genes for each group. hypergraph structure was quantified using connectivity and entropy. Clusters of highly connected genes were identified, and a wider set of transcripts were implicated as important by identifying the complete subgraph between cluster nodes and edges in the hypergraph incidence matrix. **(iii)** Biological function was assessed in two ways. GSEA was performed using genes clustered by the hypergraph or implicated by the complete subgraph in the hypergraph incidence matrix. Secondly, biological processes identified by GSEA were assessed for functional activity using hypergraphs. Hypergraphs were iterated, using subsets of genes associated with each process, and hypergraph entropy was calculated. A Bayesian modelling approach was used to detect differences in entropy distributions between processes. (**B**) **(i).** A general model of a hypergraph, shown as a three-dimensional representation of genes (coloured tetrahedra) correlating with the expression of other genes (black spheres). Shared correlations are represented by matching vertices, edges, and faces of the tetrahedra. The dimensionality of the connection between genes is defined by the number of shared correlations between those genes. ***(ii).*** A hypergraph representation of the “higher order” interactions within the transcriptome. This summary of the correlations shared between genes can be considered as the incidence matrix of a multi-dimensional network

Age-related changes in gene expression were assessed by computational analysis of transcriptomic data. Pooled samples (*n* = 5) were generated from each treatment group (SC, KD) at 5, 10, 15, 20, and 30 dpf, with three repeats per sample. Zebrafish were culled under terminal anaesthesia, and RNA was extracted from tissue anterior to the gills. Transcriptomic data were generated using Affymetrix GeneChip™ arrays. Data for all 75212 gene probes were imported into Qlucore Omics Explorer 2.2 (Lund, Sweden) as .cel files and normalised using the robust multi-array average (RMA) approach with a gene level summary. Zebrafish gene identities were assigned using Affymetrix gene definitions. Human orthologues (GRCh 38) were mapped using the *biomaRt* R-package [34]. A workflow pipeline of the transcriptomic analyses is outlined in Fig. 1a.

Unsupervised analysis of gene expression by age group was conducted to assess overall impact on the transcriptome by generating hierarchically clustered heat maps (Fig. 1ai). Standard deviation filtering (standard deviation of specific gene expression divided by maximum gene standard deviation [$s/{s}_{\text{max}}])$ was performed on the dataset to remove genes with low variance, as these were unlikely to be informative. Projection scores [41] were used to determine the threshold for filtering by calculating the maximum separation in principal component analysis (PCA).

### Hypergraph modelling

Hypergraph analysis was performed on the age-associated genes identified as above to investigate higher order interactions between target genes (Fig. 1aii), a general model of which is outlined in Fig. 1b [42]. The approach to this analysis focused on two features of the hypergraph structure. Firstly, hypergraphs form clusters of genes with higher order interactions, allowing the groups of causally associated elements to be refined from a broader set of targets [27, 42–44] as described previously [13]. Secondly, hypergraphs are not based solely on pairwise interactions, but make use of the expression patterns in the whole transcriptome to infer causal relationships between target elements. Peripheral elements of the network, representing distant action (upstream or downstream), can be identified which support these causal relationships to implicate a wider set of genes than the initial targets.

All transcriptomic analyses were performed in R (version 3.4.2). Pearson’s correlation coefficients (*r*) were calculated between age-associated genes identified by unsupervised analysis ($g,$ KD = 119, SC = 297) and the rest of the transcriptome (${g}^{c},$ KD = 75093, SC = 74915). *R*-values were binarized to generate the incidence matrix of the hypergraph (*M*). Positive and negative correlations greater than $\pm 1$ standard deviation (sd) from the mean of the R-values were assigned as ‘1’ (i.e., present) and values closer to zero were assigned ‘0’. In this way, each element ($\in )$ of *g* can be described as:

$$\in \;{\text{of}} \;g \;{\text{in}} \;M=\left\{\begin{array}{c}1, x>\pm 1sd|r|\\ 0, x\le \pm 1sd|r|\end{array}\right.$$

The resulting binary incidence matrix ($M$) was multiplied by its transpose (${M}^{t}$) to generate the reduced adjacency matrix of the hypergraph ($M.{M}^{t}$) which quantifies the shared correlations between any pair of genes ($g$). This hypergraph adjacency matrix represents the higher order interactions between pairs of genes in a manner not captured by traditional transcriptomic approaches. As a measurement of co-ordination, hypergraphs have been suggested to model functional relationships [43]. Coordination between age related genes and the rest of the transcriptome was investigated by interrogating the incidence matrix of the hypergraph and identifying a subset of edges and nodes which could form a complete subgraph (Fig. 1b). This represents the subset of the transcriptome (${\subset g}^{c})$ showing correlated expression with all the transcripts from the hypergraph central cluster ($\subset g$). This is referred to as the “peripheral” gene set (Fig. 1aii) as it represents downstream action corresponding to the hypergraph central cluster.

GWAS Catalog [45] was searched per gene to identify variants associated with cardiac or metabolic disease phenotypes. Variants with associated traits including terms ‘artery disease’,’cardiovascular disease’,’cholesterol levels’, ‘insulin levels’ or ‘myocardial infarction’ were included. A total of 14 studies were identified to establish genes we termed ‘cardio-metabolic disease GWAS genes’[46–59]. Phenome wide association of gene lists from the hypergraph analysis was conducted using PHEWAS catalogue[60].

To evaluate whether cardio-metabolic genome wide association genes were implicated in the transcriptome changes and remodelling induced by early life *grb10a* knockdown, human homologs of Affymetrix probe IDs in age-related hypergraphs were identified using biomaRt. To identify the functional importance of each GWAS gene in the SC and KD age-related transcriptome hypergraphs, the row sum of each transcript was calculated from both the binarized correlation matrices (connectivity of hypergraph incidence matrix, $M$) and the reduced adjacency matrix [61] ($M{M}^{t}$, measuring transcriptomic co-ordination by capturing the higher order interactions). Here, row sum is representative of the number of connections between the node gene and all other genes, or increased importance. Genes were ranked and then normalised. For each GWAS gene, their normalised rank was compared between SC and KD hypergraphs.

### Quantification of network coordination (Fig. 1aii)

All subsequent analyses focused on genes defined as the cluster of the hypergraph (86 genes in SC fish, 67 in KD) of 20–30 dpf zebrafish. Correlation networks model functional relationships within gene networks [62] and allow identification of clusters of highly coordinated genes [63]. Quantification of hypergraph properties (connectivity and entropy, Fig. 1aii) was performed on the hypergraph ($M.{M}^{t}$), where connectivity is the sum of connections shared by each element of the network ($g$) and entropy is the degree of disorder within the distribution of shared correlations. Entropy is positively correlated with the cellular differentiation potential [64], where a high entropy (more disorder) indicates an earlier cell lineage and multiple potential signalling pathways, and low entropy (more order) indicates a more specific differentiated function. Entropy has also been used as an index of regularity and patterning [65], and was measured using R package BioQC [66].

To assess the organization of age-associated genes within the wider transcriptome, connectivity and entropy were calculated in the 20–30 dpf cluster in each hypergraph (Fig. 1aii). As entropy scales with the size of a gene set, it was normalised for each hypergraph, resulting in a measure of the observed entropy as a proportion (ranging from 0—1) of the maximum possible entropy for a given set size, based on log values.

Robustness of the hypergraph models was assessed using 1000-fold permutation of the hypergraph incidence matrix ($M$). These permuted incidence matrices were used to generate matching reduced adjacency matrices ($M{M}^{t}$) so both the robustness of hypergraph connectivity and higher order structure could be assessed.

### Hypergraph time series

To assess the organization of the whole transcriptome over the early developmental stages of the zebrafish, a time series analysis was generated using hypergraph models (Fig. 1aii). Samples were split into age groups using a moving window approach, with each group containing fish from two sequential age points (5–10 dpf, 10–15 dpf, and so on). In this way, power is increased to be sufficient for hypergraph modelling and diminishes the impact of the individual variance at a single time point.

To form the hypergraph, the same approach was used as previously described. For the time series, 100 genes were selected at random to be the target genes. These genes were correlated against the rest of the transcriptome, with the resulting matrix binarized and multiplied by the transpose of itself; entropy was calculated on the resultant hypergraph model. This approach was iterated 1000 times, randomly selecting new target genes each iteration. Although the hypergraphs generated by this approach were consistently sized, entropy was normalized and is presented as a proportion of maximum entropy to remain consistent with the previous representation.

### Hypergraph random walk

Hypergraphs reflect associations between genes, however when derived from gene co-expression, they do not capture directionality of association. To better understand the flow between genes in the hypergraph models, a random walk approach was employed [67]. This methodology considers that, in a hypergraph, information spreading is more likely among nodes connected by the same edge, rather than transitioning between edges with every step; the result is a random walk which more completely captures the higher order effects in the hypergraph than standard graph random walks. Random walks were performed on the incidence matrices of the control and morpholino hypergraphs independently, restricted to the genes in the central cluster of each as the random walk requires that the hypergraph is not disconnected. Transition matrices were generated for each hypergraph and compared to the hypergraph adjacency matrices produced in previous analyses.

## Assessment of biological function of genes and gene pathways (Fig. 1aiii)

### Gene ontology

Gene set enrichment analysis (GSEA) was performed [68] to associate gene expression with biological processes. Genes were mapped to human orthologues using Qlucore and GSEA was performed to rank genes by age group associated ANOVA p-values. Additional GSEA was carried out through Webgestalt [69] using genes ranked by R-value, derived from a rank regression analysis of gene expression against age (Fig. 1aiii). Over-representation analysis (ORA) was used to identify gene ontology associated with unranked gene sets (Webgestalt) [69]. All gene ontology analysis used the GO Biological Process Ontology gene list [68, 70]. Primary hypergraph findings were summarised in Webgestalt using a machine learning refined interactome model termed FunMap [71].

### Hypergraph modelling of ontology

Pathways identified by GSEA were modelled using a hypergraph approach to investigate the association between each pathway in the two groups (Fig. 1aiii). Hypergraphs were generated separately for SC and KD fish using human genes associated with each pathway [72]. Pathways with fewer than 15 associated genes were removed, and all remaining gene sets were converted to zebrafish homologues using the Ensembl database (release 104) [73], queried using BiomaRt for R [34, 74].

Hypergraphs were generated on 10 genes from each pathway, iterated 1000 times. Hypergraph entropy was assessed on each iteration. A Bayesian approach was used to model the entropy distributions for each pathway to identify differences between SC and KD. This was performed using Bayesian generalized linear modelling via the R package *rstanarm* [75, 76]. Differences between SC and KD entropy distributions were calculated as a β value and significance was assigned to pathways for which the 89% credible interval of the β values did not include 0, as per established methods [77]. Positive β values represent higher entropy in SC compared to KD.

### Quantitative PCR on cardiac genes

Quantitative PCR primers amplifying markers of cardiac function were designed (Supplementary Table 1) and their efficiency validated [78]. RNA was extracted from pooled (*n* = 10) embryonic zebrafish and pooled (*n* = 3) adult (> 1 year) heart samples by QIAGEN RNeasy Lipid Tissue Extraction kit according to the manufacturer’s instructions. Samples were repeated in triplicate and tested for gene expression by qPCR (Applied Biosystems Power SyBr Green) (Agilent Technologies Stratagene Mx3005P qPCR System with MxPro qPCR Software, CA, USA) (Supplementary Table 1). β-actin was used as the housekeeping gene and the baseline Ct value was determined automatically. Relative fold change in gene expression was calculated using the ΔΔCt method according to the following equation [79]:

$$\Delta \Delta Ct={\Delta Ct}_{e3i3}-{\Delta Ct}_{SC}$$

where,

$$\Delta Ct= {Ct}_{gene \;of \;interest}-{Ct}_{housekeeping \;gene}$$

and,

$$Fold \;change= {2}^{-\Delta \Delta Ct}$$

This generates a measure of relative gene expression normalised to the expression of a housekeeping gene. The difference in relative expression between the treated and control groups is then converted into a fold change. Data were imported into GraphPad, and unpaired t-tests were performed on ∆Ct values to assess the statistical difference between samples.

### Adult body size measurements

To assess the end-stage body morphology induced by *grb10a* KD, dry mass and body length were measured in adult (18 month) zebrafish and Fulton’s condition factor was calculated. Body length was measured as the greatest straight-line distance between the snout and the end of the tail. The caudal fin was not included as fin length can be influenced by factors such as damage or variation between strains. Fulton’s condition factor was calculated according to the formula [80]:

$$K=\frac{M}{{L}^{3}}\times 100$$

where M = mass in grams and L = length in centimetres. Data are presented as the mean ± SEM (*n* = 21–34).

### Skeletal muscle and cardiac histology

Whole adult zebrafish (18 months) were embedded longitudinally in paraffin wax. 5 µm sagittal sections were taken from each tissue at a consistent depth and stained with Masson’s Trichrome (IHC World Masson’s Trichrome Staining Protocol for Collagen Fibres, Woodstock, MD, USA) to differentiate skeletal muscle (red), connective tissue (blue), and nuclei (black). Slides were scanned and visualised at 20 × magnification (3D Histech CaseViewer v2.4.0.119028, Budapest, Hungary). Skeletal muscle measurements were performed on a site lateral to the dorsal fin. The perpendicular width of individual muscle fibres was recorded and are presented as the mean of five measurements of each muscle fibre (ten fibres from five individuals, 50 fibres total).

Ventricular morphology was also analysed in ImageJ. The zebrafish heart comprises of a dense compact layer of myocardia surrounding a core of less dense spongiosa, through which the blood is dispersed. The compact layer contracts to squeeze the blood out of the spongiosa and into the circulatory system and is thus dominant in generating force [81]. Therefore, analysis of these two tissue types provides an insight into cardiac functionality. The ratio of compacta to spongiosa was calculated by measuring the area of each tissue. Data are presented as the ratio of compacta to spongiosa (%). Cardiac tissue density was calculated by restricting the region of interest to the boundary of the ventricle and calculating the total number of pixels in the image. Threshold_Colour was used to threshold the images, which were converted to 8-bit black and white images. Voxel_Counter.class was used to calculate the number of black pixels in the image. Red blood cells were digitally removed from the ventricular images prior to the calculation of density to ensure only cardiac tissue was present in the image. Cross sectional area of individual cardiac muscle trabeculae was also performed in ImageJ to test for myocyte hypertrophy following the methods of Klaiman et al. [82].

### Respirometry to assess metabolic rate and aerobic scope

Intermittent flow respirometry was used to investigate metabolic rate and aerobic scope in adult zebrafish (18 months). Sealed chambers (70 ml) were combined with recirculation loops containing an oxygen flow-through cell (Pyroscience, Aachen, Germany), paired with an optical oxygen sensor (Pyroscience, Aachen, Germany), calibrated according to the manufacturer’s instructions. A flush pump, controlled by a Cleware USB-Switch (Cleware GmbH, Germany) programmable switch and AquaResp v.3 software (AquaResp, v3, Python 3.6 [83]) was incorporated into the circuit to periodically refresh the water in the chambers (60 s flush, 30 s wait, 300 s measure). To maintain a constant temperature of 28 °C ± 0.3 °C, the respirometry system was immersed in a recirculation chamber under constant aeration. Oxygen saturation and water temperature were recorded using a FireSting (Pyroscience). Oxygen uptake rates were calculated in AquaResp using the rate of oxygen decline in the chamber during the measurement phase. Zebrafish were fasted overnight and stressed by chasing for 2 min immediately prior to the start of the first trial [84]. Maximum oxygen uptake was taken as the measurement with the greatest oxygen-uptake rate. Basal metabolic rate was calculated as the mean of the lowest 10% of the trials [85]. Aerobic scope was calculated as the difference between the maximum oxygen uptake and the basal metabolic rate. Data are presented as individual data points alongside the mean and SEM.

### Glucose metabolism

A separate group of adult zebrafish (18 months) were fasted overnight and allocated to either glucose tolerance testing or insulin sensitivity testing. All blood samples were acquired following the protocol outlined by Zhang et al. [86]. Mass, body length, and fasting blood glucose were measured prior to the start of the protocol.

Fish were anaesthetised in 0.02% MS-222, placed on their side and patted dry. A single IP injection of glucose (0.5 mg glucose/g) or glucose and insulin (0.5 mg glucose/g and 0.0075U insulin/g) was performed before recovery in 28 °C system water [87, 88]. Blood samples were taken at baseline and 30- and 120-min post-injection (glucose tolerance) or 30- and 60-min post injection (insulin sensitivity). Glucose tolerance and insulin sensitivity were based on the change in glucose over these timeframes. Individuals were culled in MS-222 before the final blood draw. Blood samples were immediately tested for blood glucose concentration (Sinocare Safe AQ blood glucose monitor, Changsha, China). Data are presented as the mean ± SEM (glucose tolerance *n* = 10, insulin sensitivity *n* = 12).

### Statistical tests

For transcriptomic analyses, rank regression (least squares method) was used to generate a linear model for each probe (the smallest degree of variance over the sample). Multiple group analysis of variance (ANOVA) was used to associate each gene ID with time dependent gene expression. Wilcoxon rank sum test (*ggpubR* package for R [89]) was used to test for differences in network topology. False discovery rate (FDR) adjustment was made using the Benjamini–Hochberg method and applied to the gene ontology analysis [90].

All other data were ROUT (Robust Regression and Outlier Removal) tested [91] for outliers and subject to D’Agostino and Pearson normality tests, with the exception of the blood glucose measurements which were measured in a validated commercial assay. Statistical tests used are described in the respective figure legends. *P*-values < 0.05 (where applicable corrected for multiple testing) were taken to indicate significance, and p-values between 0.1 and 0.05 indicating marginal significance.

## Results

### Knockdown of *grb10a* expression by splice-blocking antisense oligonucleotide

**Fig. 2:**
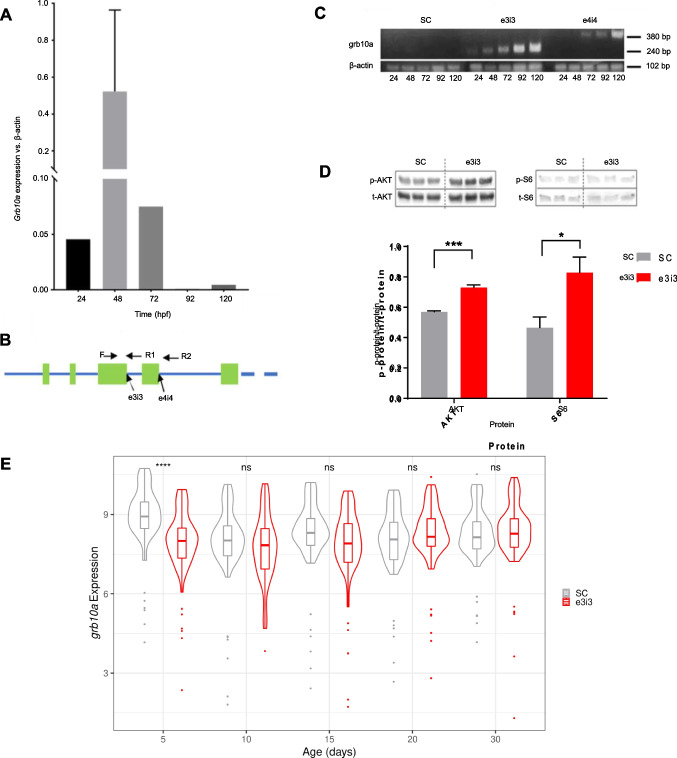
Grb10a is successfully knocked down in zebrafish injected with splice-blocking antisense oligonucleotides. (**A**) *Grb10a* qPCR of WT embryos (24–120 hpf, triplicated, *n* = 5 embryos per well). Data are shown as gene expression relative to β-actin with maximum levels at 48 hpf. (**B**) Schematic of the first five exons of the zebrafish *grb10a* gene. 5’ splice sites are highlighted with the forward and reverse primer triad indicated. **(C**) Multiplexed PCR amplification of the e3i3 and e4i4 splice site in embryos treated with either Standard Control (SC) morpholino, e3i3, or e4i4. β-actin was used as a positive control. (**D**) Western blot results of phosphorylated versus total protein ratios for two major signalling molecules of the insulin signalling pathway: AKT and S6. Quantitation using densitometry depicts mean + SEM. Activation of both proteins was found to be significantly elevated in KD zebrafish compared to SC (*n* = 3, unpaired t-test *** *p* = 0.0007, * *p* = 0.04). (**E**)*.* Expression of *grb10* mRNA throughout early life. *Grb10* expression is decreased at 5 dpf in the KD but thereafter levels in the KD are not different from those in the SC (Wilcoxon rank sum test. **** *p* = 0.00003)

As *grb10* has been linked to embryonic growth trajectory, expression was examined over the first 120 hpf. QPCR analysis of *grb10a* expression at 24-h intervals revealed a strong upregulation at 48 hpf (Fig. 2a). Expression of the *grb10* paralogue, *grb10b*, was not detectable at any time point.

To knock down *grb10a* expression, zygotes were microinjected with splice-blocking antisense oligonucleotides e3i3 and e4i4. Exon 3 and 4 donor splice sites (Fig. 2b) were targeted to confirm the specificity of the phenotype, in accordance with current guidelines [30]. Multiplexed RT-PCR amplification using primers flanking the splice sites (Fig. 2b) showed a single product of the anticipated size for e3i3 and e4i4 embryos, and no product was detected for SC embryos (Fig. 2c), consistent with retention of the corresponding intron.

To confirm *grb10a* KD induced a quantifiable impact on the downstream insulin signalling pathway, phosphorylation of key proteins was analysed by Western Blot. As shown in Fig. 2d, phosphorylated (active) versus total protein ratios of AKT and S6 were significantly elevated in *grb10a* KD zebrafish at 96 hpf compared to SC (*p* = 0.0007 and 0.04 respectively, *n* = 3), consistent with the expected impact of *grb10a* KD.

To characterise the transient nature of the morpholino knockdown, *grb10* expression was examined across the first thirty days of life. This analysis revealed that *grb10* expression was reduced in the morpholino treated group at 5 dpf (*p* = 0.00003) but returned to control levels by 10 dpf (not significant [NS]) and remained consistent throughout the juvenile phase (Fig. 2e).

### Growth trajectory and early life cardiometabolic phenotype is significantly impacted by early life *grb10a* knockdown

**Fig. 3:**
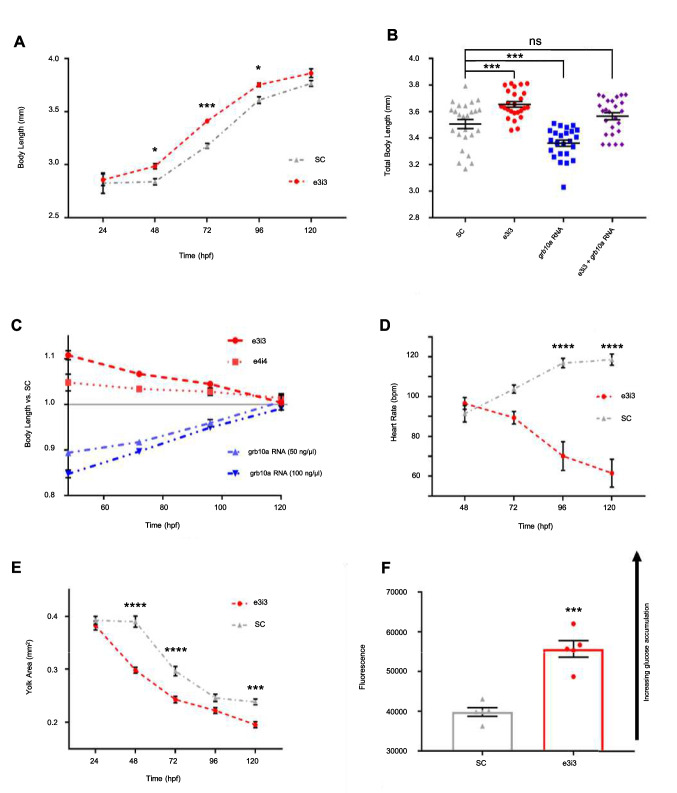
Growth and cardiometabolic phenotype are significantly impacted by early life grb10a knockdown. (**A**) Mean total body length ± SEM of SC and KD zebrafish from 24 to 120 hpf (multiple comparison 2-way ANOVA, * *p* < 0.05, *** *p* = 0.0003). (**B**) Mean total body length ± SEM and individual data points of 96 hpf zebrafish embryos (*n* = 25). *Grb10a* KD phenotype was reversed in *grb10a* overexpression zebrafish. Co-injection resulted in phenotype rescue. One-way ANOVA revealed KD zebrafish were significantly longer, while *grb10a* overexpression zebrafish were significantly smaller than SC (*** *p* = 0.0001). Rescue zebrafish were of similar length to SC (*p* = 0.38). (**C**) Mean body length measurements of the KD relative to SC ± SEM from 48 to 120hpf. (**D**) Mean heart rate ± SEM in beats per minute of SC and KD zebrafish (multiple comparison 2-way ANOVA, **** *p* < 0.0001). (**E**) Mean yolk area ± SEM of SC and KD embryos over the embryonic life stage (multiple comparisons 2-way ANOVA, *** *p* = 0.0004. (**F**) Glucose Uptake-Glo.™ Assay of 96 hpf KD and SC zebrafish, where higher luminescence indicates a greater accumulation of intracellular 2D6P. Luminescence was approximately 30% greater in KD zebrafish compared to SC (unpaired t-test, *** *p* = 0.0002)

To determine the effect of *grb10a* KD on growth, total body length was measured at 24-h intervals over the first 5 dpf. As shown in Fig. 3a*,* total body length was initially comparable between KD and SC zebrafish (2.99 ± 0.06 mm vs 2.83 ± 0.1 mm, not significant (NS), *n* = 9 and *n* = 10, respectively). Subsequently, KD zebrafish began to diverge from the SCs at 48 hpf, corresponding to the peak in *grb10a* expression observed in WT zebrafish (Fig. 3a). KD zebrafish were longer on average than SCs (3.41 ± 0.02 mm vs 3.18 ± 0.02 mm at 72 hpf, p = 0.039, *n* = 46 and *n* = 41, respectively). This phenotype was reversed in zebrafish overexpressing *grb10a,* which were significantly shorter than SC counterparts (3.36 ± 0.02 mm vs 3.51 ± 0.03, *p* = 0.001, *n* = 24 and *n* = 25, respectively), as shown in Fig. 3b. Co-injection of e3i3 and *grb10a* RNA returned body length to SC levels (3.51 ± 0.03 mm vs 3.56 ± 0.03 mm, NS, *n* = 25), confirming the validity of e3i3 induced *grb10a* KD. Moreover, *grb10a* KD induced by e4i4 (Fig. 3c) also resulted in increased body length, and the ability of *grb10a* overexpression to suppress growth was shown to be dose dependent (Fig. 3c). Intriguingly, by 120 hpf, body length converged, possibly indicating activation of compensation to regulate growth and return to an “ideal” length post-hatch.

To investigate the impact of *grb10a* KD on the developing cardiac system, heart rate was measured over the first 5 dpf. As shown in Fig. 3d, while heart rate increased slightly over time in SC zebrafish, average KD heart rate fell. By 120 hpf, mean heart rate was almost 50% lower in the KD compared to SC (61 ± 7 bpm vs 118 ± 3 bpm, *p* < 0.0001, *n* = 14 and *n* = 19, respectively).

To determine whether *grb10a* KD had an impact on metabolic rate, yolk absorption was measured to indicate nutrient utilisation and energy demand. As shown in Fig. 3e, there was initially no difference in yolk area between the groups (NS, *n* = 10). SC and KD yolk consumption began to diverge at 48 hpf (0.30 ± 0.01 mm^2^ vs 0.39 ± 0.01 mm^2^, *p* < 0.0001, *n* = 18 and = 19, respectively). The greater yolk consumption observed in the KD fish suggests an elevated metabolic rate. To support this conclusion, a Glucose Uptake-Glo™ Assay was performed to compare the rate of glucose uptake, measured by 2D6P content. As shown in Fig. 3f*, *2D6P accumulation was significantly higher in KD zebrafish compared to SC, an increase of almost 30% (*p* = 0.0002, *n* = 5), indicating glucose uptake was elevated. These growth and metabolic findings are consistent with the role of *grb10a* as a negative regulator of growth and insulin signalling pathway [92].

### Early-life *grb10a* knockdown persistently dysregulates age-associated gene expression

In advance of transcriptomic analyses, tissue types represented in the dataset were quantified by assessing patterns of gene expression from an independent zebrafish scRNA-seq dataset annotated to tissue level [40]. As anticipated, the 5–30 dpf zebrafish samples most closely resembled the transcriptome of mid-hindbrain tissue (> 88%), with additional contribution from other neural tissues as well as cephalic and skeletal muscle (Supplementary Table 2).

**Fig. 4:**
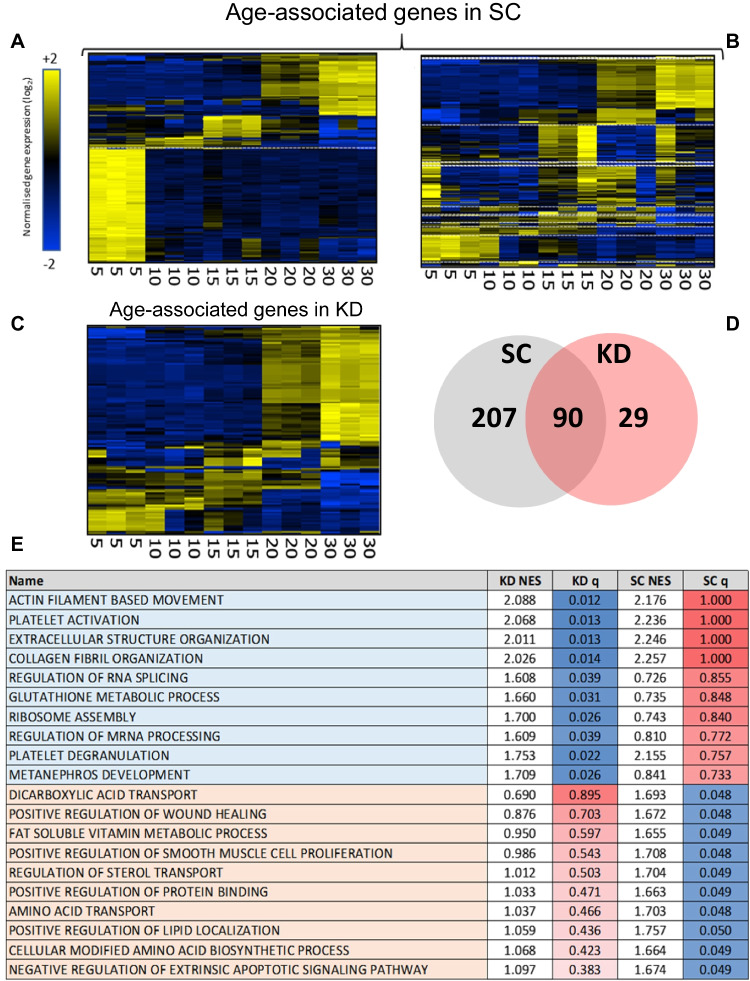
Transcriptomic analysis of standard control and grb10a knockdown gene expression over the first 30 dpf. Hierarchically clustered heat maps of gene expression generated from an Affymetrix GeneChip™ Zebrafish Genome Array of SC and KD zebrafish RNA, taken at 5, 10, 15, 20, and 30 dpf. (**A**) Expression of age-related genes in SC segregates into three clusters in SC zebrafish. (**B**) Clustering of the same age-related genes (identified in **A**) is disrupted in KD. Notably, the cluster of genes expressed at 5 dpf in SC are expressed at a variety of time points in KD. (**C**) Analysing the KD dataset independently highlights a set of genes with age related gene expression which fall into two clusters. (**D**). Venn diagram of age associated genes identified separately in SC and KD demonstrates that only a small proportion of SC age associated genes (30%, 90/297) remained age associated in KD. (**E**) Gene set enrichment analysis, using the GO Biological Process Ontology gene list, of the age related genes in the SC and KD datasets. The top 20 most enriched pathways with differential expression are included here, with associated normalised enrichment scores and *q*-values

To understand whether the changes observed in *grb10a* KD embryos were coupled with lasting changes in gene expression, the transcriptomic landscape of SC and KD zebrafish was investigated over the first 30 dpf. Unsupervised hierarchical clustering, standard deviation filtering, and maximised projection scores (MPS) were used to define a set of genes with strong age-association in the SC zebrafish (297 genes, MPS = 0.43). These genes fell into four distinct clusters, associating with 5, 10, 15, and 20–30 dpf (163, 15, 32, and 87 genes respectively) (Fig. 1ai and Fig. 4a).

Upon examining expression of the same genes in KD zebrafish, the clusters of co-expressed genes identified in the SC animals (Fig. 4a) was not present in the KD zebrafish (Fig. 4b). While the clustering was largely conserved in the latter three clusters (10 dpf– 14/15, 15 dpf– 29/32, and 20–30 dpf– 74/87 genes), genes strongly associated with 5 dpf in the SCs were generally expressed at different time points in the KDs (dotted white lines, Fig. 4a and Fig. 4b), with a mapping of only 38/163 genes (23%). Notably, five genes associated with 5 dpf in SC zebrafish mapped to 20–30 dpf in the KD. This suggests that the expression of genes usually associated with early larval development, is instead associated with the late-juvenile stage in KDs. Human orthologues of these dysregulated genes include *DGAT2* (fatty acid metabolism), *GAMT* (energy storage, muscle contraction, and fatty acid oxidation), and *PDIA2* (oxidative stress).

As the SC gene clusters were disrupted in the KD dataset, unsupervised analysis of the KD data was performed to identify the subset of age-associated genes in the KD zebrafish. 119 genes were identified (MPS = 0.37) which segregated into 5–15, 15–30, and 20–30 dpf (37, 16, and 66 genes) (Fig. 4c). Although 75% (90/119) of age associated genes in the KD were shown to have been age associated in SC, only a small proportion (30%, 90/297) of genes which were age associated in SC were also age associated in KD (Fig. 4d).

To assess functionality of the identified age-related genes (Fig. 1ai), Gene Set Enrichment Analysis was performed for genes identified in both the SC and KD datasets. Functionality conserved between the SC and KD is described in Supplementary Table 3. Dissimilar pathways (Fig. 4e) included several actin and collagen related pathways, and extracellular structure and RNA processing, which were age-related in KD but not SC zebrafish. Conversely, several metabolic pathways were age-associated in SC zebrafish but not in KD animals. This dysregulation of age-related gene expression in the KD zebrafish implies early life *grb10a* KD induces remodelling of the transcriptome.

**Fig. 5:**
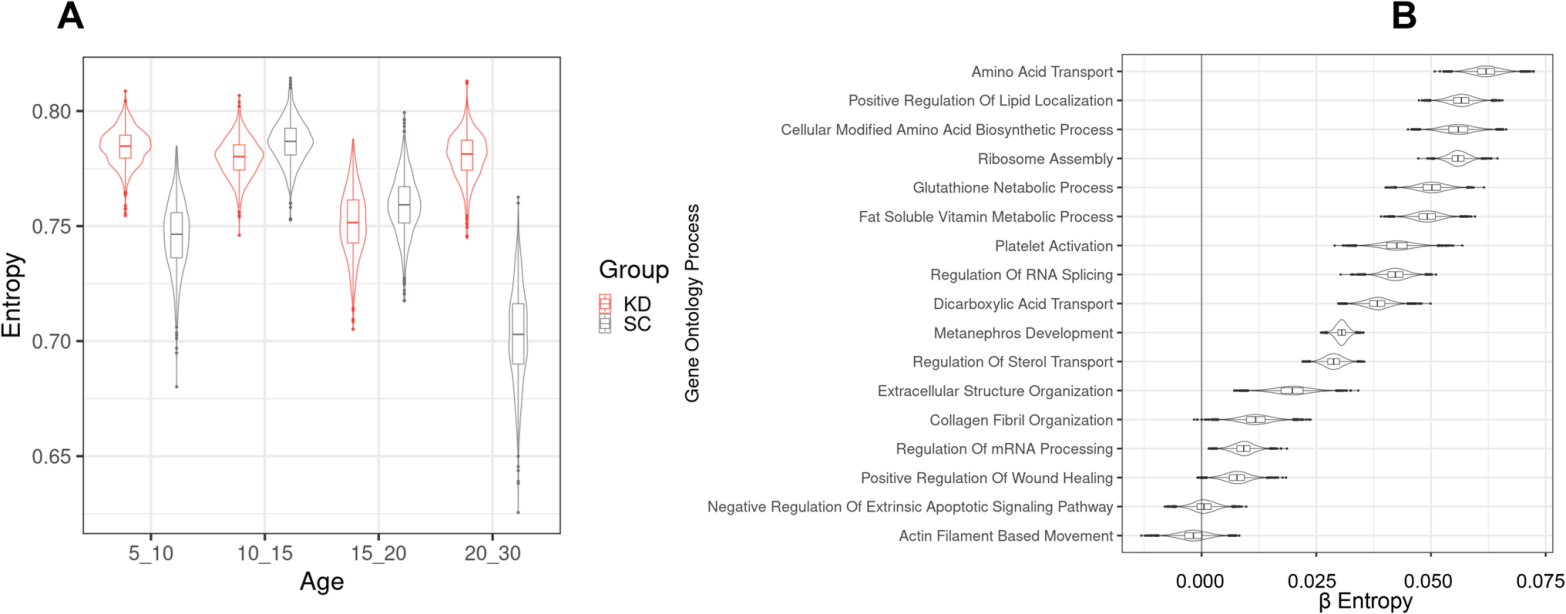
Hypergraph entropy in the transcriptome reveals differences over time and between functions. (**A**) hypergraphs were iterated using randomly selected genes and a moving window approach to assess change in transcriptomic entropy over time. SC and KD are significantly different at all time points (*FDR* < *2.4* × *10*^*–11*^* ANOVA with Tukey post-hoc)* though the largest differences exist at 5–10 dpf (*fold change* = *0.052*) and 20–30 dpf (*0.11*). (**B**) hypergraphs were iteratively generated from genes attributed to each pathway and entropy was modelled across the two treatment groups. β values represent the difference in entropy between SC and KD, with a β value of 0 indicating no difference between groups. Positive β values represent higher entropy in SC compared to KD. Ontology classes are considered to be significantly different if the 89% CI of β values does not include 0. *Actin filament-based movement* and *negative regulation of extrinsic apoptotic signalling pathway* were the only two pathways with no difference identified between the groups

We next assessed transcriptomic organisation using hypergraphs. We discerned a significant increase in the entropy of the transcriptome in KD compared with SC at 5-10dpf and 20-30dpf (FDR < 2.4 × 10^–11^) (Fig. 5a). Entropy is a measure of information content and serves as an indicator of “disorder”, such that lower entropy (more order) describes a network with little crosstalk and a more discrete function [64, 93], while a network with greater entropy (more disorder) has increased crosstalk and pleiotropic functions. This demonstrates that significant differences in the organisation of the transcriptome occur in the early-larval phase as a result of the *grb10a* KD with a further reorganisation during later development.

By investigating pathways enriched in the age-related cluster (Fig. 4e), we were able to demonstrate their contribution to the organisation of the transcriptome by measuring the difference in entropy for each pathway between KD and SC (Fig. 1aii). Most of these pathways had higher entropy in SC than KD (Fig. 5b). The greatest differences in activity between the KD and SC were in *Positive Regulation of Lipid Localization*, *Amino Acid Transport* and *Cellular Modified Amino Acid Biosynthetic Process*. This indicates that entropy and gene coordination at the whole transcriptome level (higher in KD) are different at the level of these pathways (higher in SC).

By employing a random walk, transition between nodes in the network can be modelled probabilistically. Although this does not capture the mechanistic interactions between genes, it reflects flow and coordination in the system, to more closely reflect the causality of a molecular cascade. The structure of the random walk transition matrix was strongly related to the structure of the hypergraph adjacency matrix, as measured by correlation between the two (control r = 0.63, morpholino r = 0.84) (Supplemental Fig. 2). This demonstrates that although the hypergraph adjacency matrix does not consider directionality of association, it nevertheless captures the hierarchical structure of molecular cascades as inferred from random walks.

### Early-life *grb10a* knockdown severely disrupts coordination within larval gene clusters

**Fig. 6:**
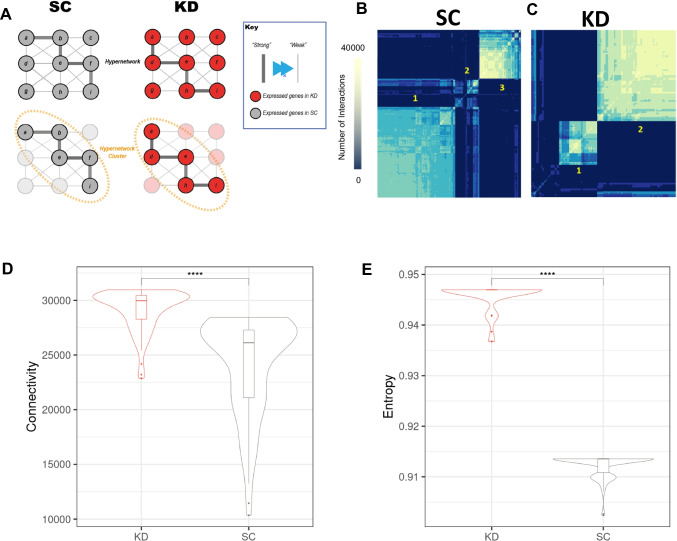
The coordination of age associated genes is altered by grb10a KD, as assessed by hypergraph analysis (**A**) hypergraphs form clusters of genes with higher order interactions between them; this allows us to refine groups of causally associated elements from a broader set of targets. (**B**) hypergraph analysis of the identified age-associated genes formed three such clusters of genes in SC zebrafish, corresponding to [1] 5 dpf, [2] 10–15 dpf, and [3] 20–30 dpf. (**C**) Two clusters were defined in the KD data, corresponding to [1] 5–15 dpf and [2] 20–30 dpf. (**D**) *and* (**E**) Violin plots of connectivity (left) and entropy (right) in the 20–30 dpf cluster. The KD transcriptome was more connected with higher entropy compared to SC, suggesting a more diverse set of connections are made by age associated genes (Wilcoxon Rank sum **** *p* < 0.0001)

To investigate the co-ordination of the whole transcriptome with age-associated gene expression, hypergraph models were constructed for the SC and KD datasets based on the gene sets identified in the cluster analysis (297 and 119 genes respectively) (Fig. 1aii). This approach has been used to elucidate clusters of causally associated genes with co-ordinated regulation [44] (Fig. 6a). Figure 6b, c demonstrates this clustering, where colour intensity represents the number of shared correlations between each gene pair. In SCs, genes segregated into three highly connected groups, associated with 5, 10–15, and 20–30 dpf (161/297, 50/297, and 86/297 genes) (Fig. 6b). In KD animals, however, genes segregated into only two groups, either 5–15 dpf or 20–30 dpf (31/119 and 65/119 genes) (Fig. 6c). Notably, the large group of co-ordinated interactions at 5 dpf was absent in the KD dataset, with a combined group of genes correlating with 5–15 dpf identified instead.

### *Grb10a* knockdown is associated with increased connectivity and crosstalk between age related genes

To quantify these differences in the co-ordination of the transcriptome, two network topology parameters were used: connectivity and entropy (Fig. 1aii). Hypergraph connectivity quantifies the number of higher order interactions within the transcriptome and entropy is a measure of ‘disorder’, as described above, with both related to function [43, 44].

Changes in connectivity (Fig. 6d) and entropy (Fig. 6e) were calculated for genes identified as active at 20–30 dpf by the previously described analyses. The impact of KD on the co-ordination of age-associated genes in the network was identified by comparing the connectivity and entropy of SC and KD datasets. To provide a comparison between age-associated genes and genes associated with other functions, connectivity and entropy were also calculated for randomly selected gene sets (Supplemental Fig. 1).

Genes associated with 20–30 dpf were more highly connected and less entropic (more ordered) than random genes in both the KD and SC datasets (*p* < 2.2 × 10^–16^). Comparison of age associated genes in KD and SC revealed a higher connectivity (1.20-fold, *p* < 2.2 × 10^–16^) (Fig. 6d) and a higher entropy (1.05-fold, *p* < 2.2 × 10^–16^) in the KD (Fig. 6e) This suggests age-associated genes in the KD zebrafish transcriptome have more interactions and more crosstalk (less ordered) than in the SC.

### Pathways associated with transcriptome-wide remodelling support an alteration in cardiometabolic phenotype

**Fig. 7:**
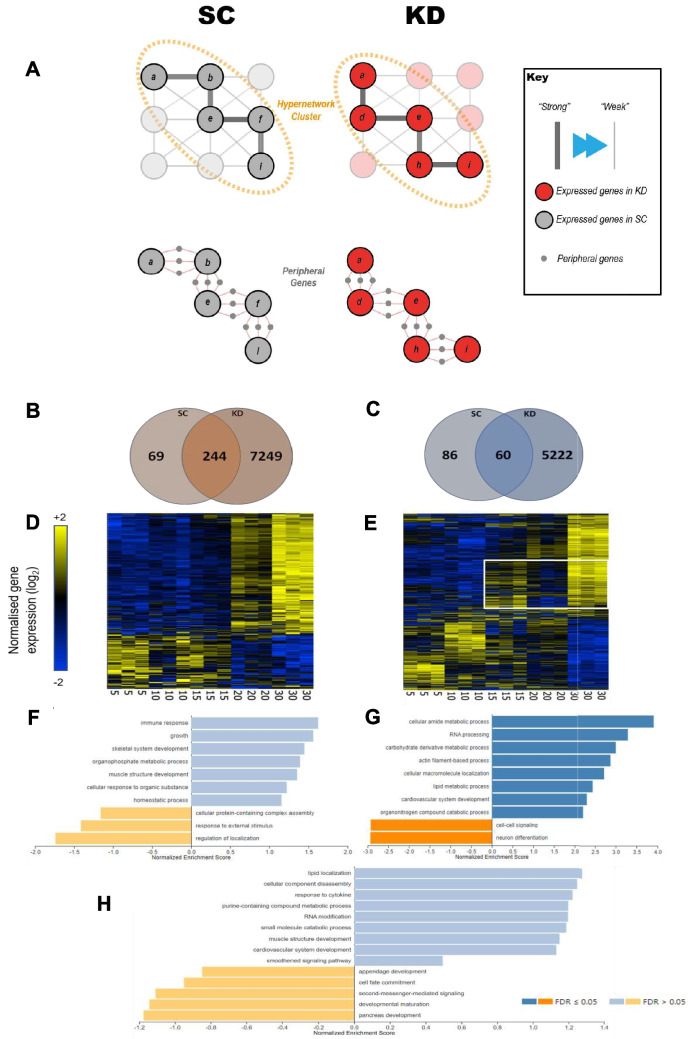
Analysis of the set of genes in the wider transcriptome shows a 27.8-fold increase in the KD ZF. (**A**) Identification of peripheral elements underpinning the hypergraph implicates a wider set of genes than the initial targets. A summary of those peripheral elements is presented here. (**B, C**) Venn diagrams of genes positively (**B**) and negatively (**C**) correlating with age. (**D, E**) Hierarchically clustered heat maps of gene expression of the genes identified in the wider transcriptome. Gene expression in the standard control (**D**) cluster into two age related groups, whereas expression in the knockdown (**E**) show significant dysregulation. (**F, G**) Gene set enrichment analysis (GSEA) ranked by *R*-value of rank age regression in the standard control (**F**) and knockdown (**G**). (**H**) GSEA ranked by *R*-value of rank age regression of the cluster of genes in the white box in (**E**)

Having identified a central set of age-related genes, a broader set of genes, which coordinate with these genes, represent upstream or downstream interactions associated with this central core of the hypergraph (Fig. 1aii). This broader set of genes are termed “peripheral genes” as they represent changes in expression consequential but distant from the hypergraph cluster (Fig. 7a). 12775 (KD) and 460 (SC) genes were defined in this set from the 20 to 30 dpf hypergraph clusters described previously, all significantly associated with age (rank-regression: KD q < 1.50 × 10^–6^, SC q < 4.44 × 10^–2^; Supplementary Table 4). 28 times more peripheral genes were implicated in the KD. Both datasets showed a skew towards a positive association with age (68% SC vs 59% KD) (Fig. 7b*, *Fig. 7c). The overlap in gene expression between the two datasets was 244 (77.9% of SC) and 60 (41.1% of SC) in the positively and negatively correlated sets, respectively. Thus, genes in the wider transcriptome with co-ordinated expression at 30 dpf in SC zebrafish demonstrated a similar pattern of expression in KD (Fig. 7d vs. e). However, the inverse is not true, as genes with co-ordinated expression at 15–30 dpf in the KD zebrafish did not show the same pattern in the SC (Fig. 7e vs. d).

To assign biological function, GSEA on the peripheral genes was performed as previously described (ranked by R-value, top 100 pathways with weighted set cover) (Fig. 1aiii). Results of this ontology analysis are outlined in Fig. 7f (SC) and Fig. 7g (KD). The two datasets featured distinctly different regulated pathways. RNA processing, a variety of metabolic pathways, and cardiovascular development featured in the KD dataset, whereas growth and immune signalling were featured in the SC dataset.

**Fig. 8:**
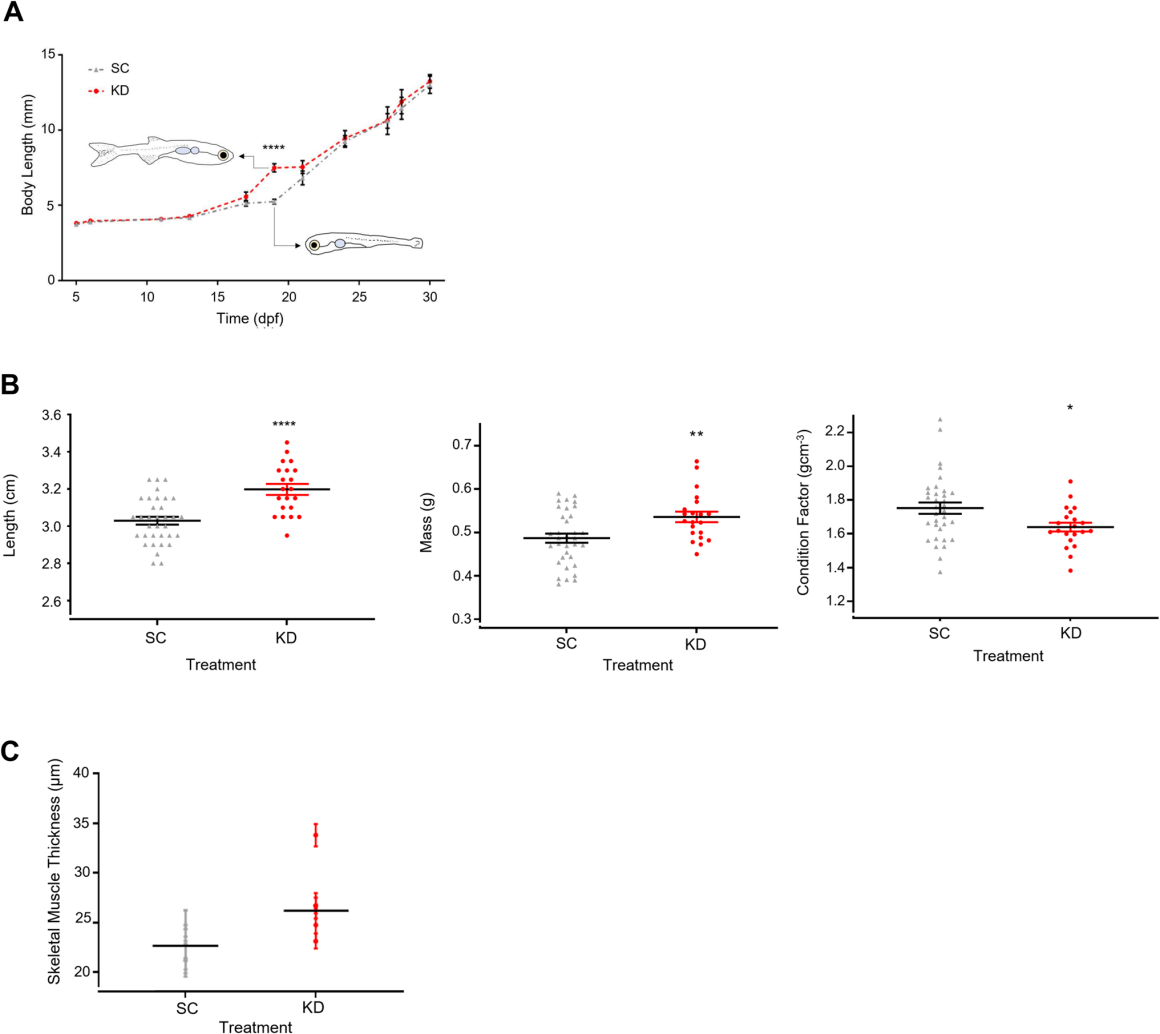
Growth in juvenile Zebrafish and adult size and skeletal muscle thickness at 18 months. (**A**) Mean total body length of SC and KD zebrafish up to 30 dpf. Following the embryonic growth spurt, KD zebrafish experienced an additional period of rapid growth between 15 and 20 dpf (unpaired t-test, **** *p* < 0.0001). Typical morphology for body length at 18 dpf is included for reference. (**B**) Individual total body length (left panel), mass (middle panel), and condition factor scores (right panel) for 18-month KD and SC zebrafish (*n* = 21–24). Length and mass were significantly higher in the KD (unpaired t-test, **** *p* < 0.0001, ** *p* = 0.005), while condition factor was significantly lower (* *p* = 0.02), indicating KD zebrafish have leaner bodies. (**C**) Thickness of 10 skeletal muscle fibres per fish (*n* = 5) stained with Masson’s Trichrome. Fibre thickness was marginally higher in KD compared to SC zebrafish (repeated measure ANOVA, *p* = 0.06). Data are presented as overall group mean and as mean ± SEM for each fish

A distinct pattern of gene expression was identified in the group of genes dysregulated at 20–30 dpf in the KD dataset (white box, Fig. 7e). These genes were upregulated at 15 dpf, downregulated at 20 dpf, and re-upregulated at 30 dpf. This subgroup of 3460 genes (Supplementary Table 5) was associated with an enrichment of gene ontologies including lipid metabolism, muscle development and cardiovascular system development (Fig. 7h) and was synchronous with the spike in growth identified in Fig. 8a. Specific pathways following this pattern of expression included cardiovascular system development, muscle structure development, and developmental maturation.

### The impact of early life *grb10a* KD persists into adulthood

As *grb10a* KD significantly impacted early-life growth, metabolism, and cardiovascular development and was associated with remodelling of the transcriptome between 5 and 30 dpf, investigation was conducted into the phenotypic differences in older zebrafish.

Total body length measurements up to 30 dpf are shown in Fig. 8a. Body size was significantly elevated in the KDs compared to SCs during larval development between 15 and 20 dpf (30% increase, 7.49 ± 0.27 mm vs 4.32 ± 0.16 mm, *p* < 0.0001, *n* = 10). This corresponded to the cluster of dysregulated genes identified by hypergraph modelling (Fig. 7h and white box in Fig. 7e). The growth profile of the KDs was shifted to an earlier age compared to the SC, suggesting a faster rate of maturation. The GSEA highlighted a reduction in the activity of developmental maturation pathways (normalised enrichment score < 1) in SCs vs. KDs, which was reflected in the growth rate at approximately 20 dpf. This time frame is also characterised by rapid morphological changes as the dorsal, anal, and pelvic fin buds develop and mature and the larvae begin to resemble an adult zebrafish [94].

Later-life body length and mass measurements were recorded in 18-month-old zebrafish to investigate the lasting impact of *grb10a* KD on the phenotype. Final body length (Fig. 8b*.*) was higher, with KDs 0.17 cm longer than SCs (32 ± 0.03 cm vs 30 ± 0.02 cm, *p* < 0.0001). KD fish were also approximately 10% heavier (0.54 ± 0.01 mg vs 0.49 ± 0.01 mg, *p* = 0.005). Fulton’s condition factor (an indicator of body condition) was lower in the KDs (1.64 ± 0.03 vs 1.75 ± 0.03, *p* = 0.022) despite the increase in mass, suggesting a “leaner” phenotype. To equate this to differences in body composition, skeletal muscle fibres, isolated from the base of the dorsal fin, were sectioned and stained with Masson’s Trichrome (Fig. 8c). The average muscle fibre diameter was approximately 20% greater in the KDs, which was marginally different between groups (*p* = 0.06).

**Fig. 9:**
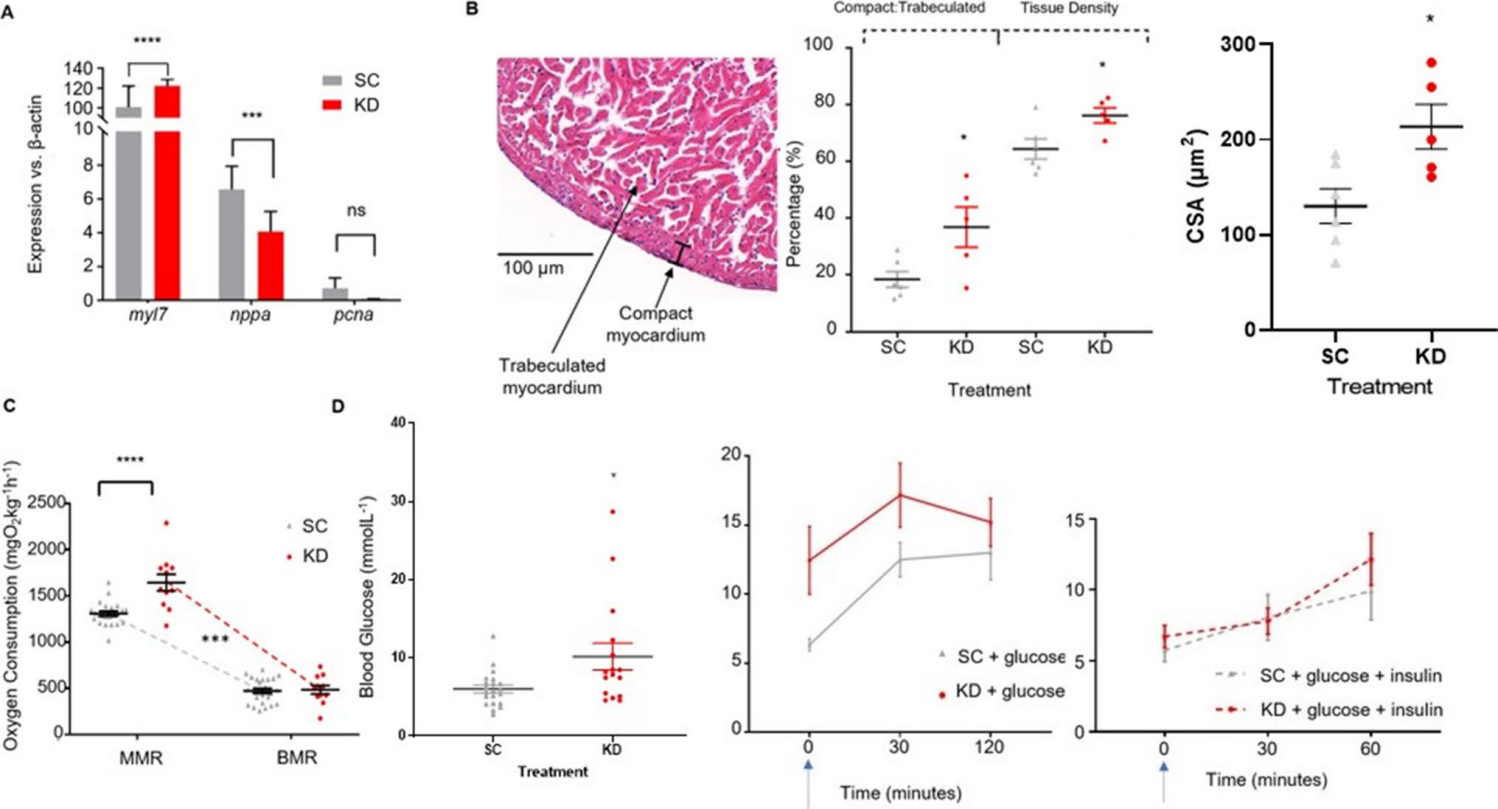
Cardiac gene expression and Cardiometabolic function in 18-month-old adult Zebrafish. (**A**) qPCR results of three genes associated with cardiac performance in adult cardiac tissue, relative to β-actin. Myl7 expression was significantly elevated in KD zebrafish (unpaired t-test, **** *p* < 0.0001) while nppa expression was significantly down regulated (*** *p* = 0.001) compared to SC zebrafish. There was no significant difference between the expression of pcna (*p* = 0.3). (**B**) Ventricular morphometrics obtained by Masson’s Trichrome histology comparing KD and SC compacta thickness, tissue density, and fiber cross sectional area. The compacta layer was significantly thicker (unpaired t-test, * *p* = 0.03) and overall tissue density was higher (* *p* = 0.03) in the KD zebrafish, and the fiber cross-sectional area (CSA) was increased (* *p* = 0.02) (*n* = 5–6). (**C**) Maximum (MMR) and Basal (BMR) oxygen uptake rate of adult (18 month) zebrafish, adjusted for body mass. BMR was comparable between the two groups (unpaired t-test, NS), while MMR was greater in the KD zebrafish (**** *p* < 0.0001), resulting in a greater aerobic scope (dotted line, unpaired t-test, *** *p* = 0.0007). (**D**) Fasting Glucose concentrations (left panel), glucose tolerance testing (centre panel) and insulin sensitivity testing (right panel) in adult (18 month) KD and SC zebrafish. Fasting glucose was higher in the KD (*n* = 16) than in the SC (*n* = 21) (unpaired t-test, *p* = 0.01). There were marginally higher glucose concentrations in the KD zebrafish in the glucose tolerance test (repeated measures ANOVA, *p* = 0.07) but both KD and SC zebrafish responded similarly to insulin (repeated measures ANOVA, NS). Treatment started immediately after the first measurement. Data are presented as mean values ± SEM

The impact on the heart was assessed by qPCR and histology. As shown in Fig. 9a, *myl7* expression (an index of muscle mass and hypertrophy) in the heart was over 20% greater in KDs (*p* < 0.0001, *n* = 3), while *nppa* expression (activated in response to ventricular stress during hypertrophy and heart failure [95, 96]) was reduced by approximately 40% (*p* = 0.0012, *n* = 3). There was no difference in *pcna* expression (proliferating cell nuclear antigen), suggesting there was no difference in proliferation in the cardiac tissue. These observations were confirmed in the age-related gene expression from the transcriptomic data (Supplementary Table 6). To support these findings, ventricular morphology was also assessed. As shown in Fig. 9b, the ratio of compact myocardial layer to trabeculated was significantly greater in the KD zebrafish (37% vs 19%, *p* = 0.03). This was coupled with an overall increase in tissue density (76% vs 64%, *p* = 0.03). The cross-sectional area of cardiac muscle myocyte bundles was also significantly greater in KD zebrafish compared to SC counterparts, with an approximate 50% increase in bundle cross sectional area (214 ± 24 µm^2^ vs 130 ± 18 µm^2^, *p* = 0.0195).

To determine whether metabolic rate was elevated later in life, oxygen uptake in adult (18-month) zebrafish was also investigated. Maximum oxygen uptake, achieved following exhaustive activity, was elevated by ~ 25% in KDs, as shown in Fig. 9c (1645 ± 90 mgO_2_kg^−1^ h^−1^ vs 1309 ± 27 mgO_2_kg^−1^ h^−1^, vs *p* < 0.0001). There was no significant difference in basal metabolic rate (486 ± 47 mgO_2_kg^−1^ h^−1^ vs 474 ± 28 mgO_2_kg^−1^ h^−1^). Consequently, aerobic scope was greater in the KDs (1158 ± 101 mgO_2_kg^−1^ h^−1^ vs 810 ± 43 mgO_2_kg^−1^ h^−1^, *p* = 0.0007).

As grb10a is involved in regulating the insulin signalling pathway, glucose tolerance and insulin sensitivity tests were performed on adult zebrafish (18 months) to determine whether there was a lasting biological impact. Fasting blood glucose (Fig. 9d) showed a significant difference between SC and KD fish, higher in KDs (10.2 ± 0.9 mmol/L vs 6.0 ± 0.5 mmol/L, *p* = 0.01). Glucose tolerance testing showed marginally higher glucose levels in the KD zebrafish at *p* = 0.07, while both groups produced a similar response to insulin administration.

### Cardiometabolic genetic risk is functionally linked to the *grb10a* KD age-related transcriptome

There were 29 shared age-related genes that overlapped between SC (297 genes) and KD (119 genes) hypergraphs (Fig. 6). A total of 19 variants were identified to establish a group of 6 genes we termed ‘cardio-metabolic disease GWAS genes’ (Supplementary Table 7). Recently, 170 serum protein quantitative trait loci have been identified for cardiometabolic trait loci [97] representing 0.89% of the protein coding genome. The observation of 6 cardio-metabolic disease genes out of 29 represents a highly significant enrichment (Fisher’s Exact Test *p*-value = 0.0006 [2-tailed], odds ratio = 29).

**Fig. 10:**
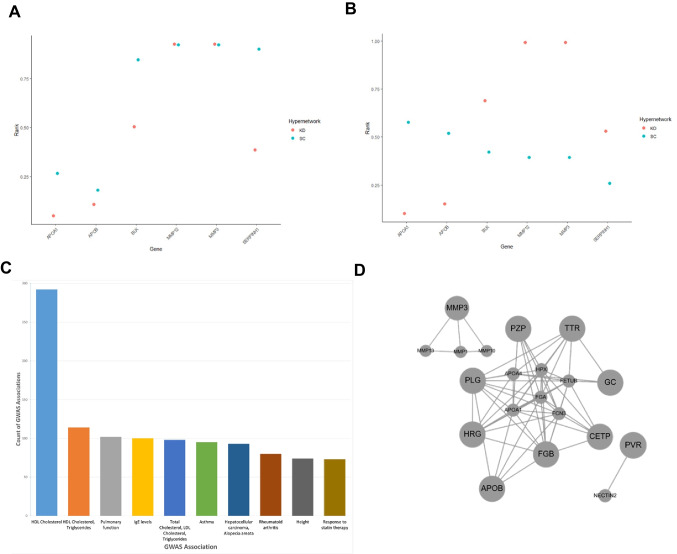
Genes associated with cardio-metabolic diseases. Cardio-metabolic disease GWAS genes ranked by normalized row sum in the age-related transcriptome hypergraphs of control and GRB10 knockdown zebrafish. Six genes out of 29 differentially expressed genes were present in both the SC and KD were shown to be related to cardiometabolic health. **A**) The impact of these genes on hypergraph connectivity and,** B**) higher order interactions in the transcriptome hypergraph were measured and ranked using a normalized ranking score, higher value indicates greater number of interactions in the transcriptome and implies greater impact on function by increased cross talk between biological pathways. **C**) Human Genome Wide Association Study (GWAS) relationships identified by phenome wide association using the fourteen human orthologous genes present in overlap between SC and KD hypergraphs. Identified using PHEWAS catalogue. **D**) Interactome model of the fourteen human orthologous genes present in overlap between SC and KD hypergraphs, demonstrating coherency and implying co-ordination

Ranked row sums of the hypergraph incidence matrix (representing the hypergraph connectivity) were compared for each GWAS gene in SC and KD age-related transcriptome hypergraphs (Fig. 10A). Four GWAS genes, *APOA1*, *APOB*, *BLK* and *SERPINH1*, were ranked lower in the KD hypergraph compared to the control hypergraph. This suggests these cardiometabolic disease associated genes are less functionally important after the transcriptome changes induced by *grb10a* knockdown in early life. Other cardiovascular and metabolic GWAS genes, *MMP3* and *MMP12,* did not show a difference in rank between the control and knockdown model, suggesting the transient *grb10a* knockdown does not affect the functional importance of these genes in the transcriptomic network.

We assessed the impact of higher order interactions in the transcriptomic hypergraph independently using the reduced adjacency matrix row sums. This was performed for each of the identified cardio-metabolic disease GWAS genes (Fig. 10B). This analysis demonstrated persistent differences in higher order interactions between KD and SC with four of the genes more highly ranked in KD (*BLK*, *MMP12*, *MMP3* and *SERPINH1*) and two of the genes more highly ranked in SC (*APOA1* and *APOB*).

Permutation of both the hypergraph incidence matrix and hypergraph reduced adjacency matrix was performed 1000 times and used to show robustness of the network ranks associated with the six human orthologous genes (Supplementary Fig. 3).

To examine wider genomic relationships, we examined phenome wide association for the 14 human orthologous genes that mapped to the 29 shared age-related genes that overlapped between SC and KD hypergraphs (Supplementary Table 8). This analysis highlighted a range of related genome wide associations studies (Fig. 10C) that mapped mainly to pathways related to cholesterol metabolism and lung function (7.4 × 10^–10^ < *p* < 0.001, Supplementary Table 8). An inferred interactome model of these 14 human genes demonstrated coherence of transcriptomic action (Fig. 10D) and highlighted overall impact on extracellular matrix, protein-lipid complex and collagen pathways (Supplementary Table 9).

We demonstrated by measuring phosphorylated protein that AKT and S6 were significantly elevated in *grb10a* KD zebrafish indicative of modulation of IGF and Insulin pathways (Fig. 2C). We screened the SC and the KD hypergraphs (Fig. 6) for evidence of phospho-ERK activity by mapping the associated genes to ERK targets [98]. A primary ERK signalling target is *FOS* and this featured in the SC hypergraph but was absent in the KD hypergraph. FOS is a primary target of ERK signalling and a transcription factor associated with cell proliferation and differentiation [99] (Supplementary Table 10).

## Discussion

The first key finding from this study is that grb10a regulates embryonic growth in zebrafish, consistent with its role in mammalian embryogenesis [17, 25, 26], and that knockdown in the early embryo is sufficient to have an impact on downstream pathways. This is consistent with its role as a negative regulator of insulin signalling [16, 100, 101]. Knockdown resulted in lower *grb10* mRNA levels at 5dpf, but no differences in g*rb10* mRNA from 10–30 dpf. There was upregulation of signalling molecules in the insulin pathway downstream of grb10a at 4 dpf, which was coupled with significant changes to the early phenotype, including elevated growth rate, increased metabolism, and reduced heart rate between 1 and 5 dpf. The observed changes in phenotype coincided with the peak in *grb10a* expression, reinforcing the role of *grb10a* as a coordinator of embryonic growth and development. The elevated growth coincided with higher metabolic rate, consistent with an increase in energy demand due to a larger population of highly proliferating cells. Changes in heart rate appeared slightly later in development, around 72 hpf, but still support the conclusion that grb10a has a role not only in coordinating growth and metabolism, but also in cardiac function.

This study provides evidence for a compensatory growth mechanism activated during early larval development, which regulates adherence to an “ideal” body length. The increased embryonic growth in the KD is followed by a decreasing growth rate at 4 and 5 dpf, with body length not different between groups at 5 dpf. The altered early-life growth trajectory may serve as a basis for the long-term changes to the organism. At 17 to 21 dpf, there is a divergence in the growth trajectories with the KDs showing a period of accelerated growth and increased physical maturity compared to the SCs. The growth trajectories then converge again. However, at 18 months the adult KD zebrafish are longer and heavier than the SCs. This increased size in the KDs associated with changes in cardiac ventricular morphology, metabolic rate and glucose metabolism occurred despite no further manipulation to the organism.

Furthermore, this study has shown that early-life disruption in the expression of grb10 can result in long-term remodelling of the transcriptome. This highlights the importance of regulated control during embryogenesis and the significant impact small changes, such as an increase in growth, can have on the developing organism. Age-associated genes in both the SC and the KD transcriptomes showed increased connectivity and decreased entropy compared to non-age associated genes. This demonstrates that the genes identified as age-associated are better connected to one another and more ordered in their interactions than randomly selected genes. Entropy and connectivity were both higher in KD than SC, and, while both metrics were significantly different between age-associated and random genes, the greater increase in connectivity and smaller decrease in entropy in the KD transcriptome further demonstrates the KD transcriptome is organised in a different way to that seen in the SC with a larger number and a wider variety of interactions.

Notably, gene expression at 5 dpf was significantly dysregulated in the KD zebrafish compared to the SCs. An early transition in gene expression identified in the SCs, occurring between 5 and 10 dpf, likely corresponding to a shift away from early developmental pathways, was lost in KD zebrafish. This loss was reflected both in the age-related genes and the coordination of the wider gene set. This fundamental difference in the transcriptome during larval development was particularly notable in a subset of genes with fluctuating gene expression in the KD zebrafish. Genes associated with cardiovascular development, muscle development, and developmental maturation showed a distinct pattern of upregulation at 15 dpf, downregulation at 20 dpf, and upregulation again at 30 dpf, coinciding with the spike in growth in the larval zebrafish. This pattern occurred beyond the impact of the morpholino, implying adaptive impact, and may contribute to the left-shift in growth rate observed in the KD zebrafish, which is mirrored in children experiencing early puberty [102]. This developmental period is also characterised by rapid morphological changes during which the larval zebrafish begins to resemble the adult. As illustrated in Fig. 8a, zebrafish of the size of the KD at 18 dpf (7.5 mm) typically display complete flexion of the caudal fin, and fin rays are rapidly developing in the caudal, dorsal, and anal fins. The first forking event is also complete in the caudal fin, and the pelvic fin buds and second lobe of the swim bladder have formed [94]. In contrast, the SC zebrafish, at only 4.3 mm, still typically resembles the larval form. Flexion of the caudal fin has not begun, and the fin buds have not begun to develop (Fig. 8). This major difference in growth and development in the KD is consistent with accelerated physical maturation as seen in an early human puberty.

This model of embryonic growth perturbation may also yield significant insights into the propensity for early growth disruption to correlate with increased risk of cardiovascular and metabolic disease in later life [103–106]. It is widely accepted that many disorders are likely to have their origins during embryonic development [107–109], but the mechanisms involved are not fully understood, and targeted *in vivo* research is lacking. Mammalian models have been used to investigate the immediate impact of embryonic growth disruption [110–112], findings which are replicated in this study. However, longitudinal studies are absent from the literature, and little research has been conducted into the whole-life significance of early growth disruption. Multiple distinct growth trajectories can yield similar birth weights, including catch-up and catch-down growth [103] (both of which were achievable by modulation of *grb10a* expression). Catch-up and catch-down growth have been reported to correlate with increased risk of chronic health disorders [106, 113, 114], and as *grb10a* modulation is sufficient to alter embryonic growth trajectory, metabolic rate, and heart rate, the model generated in this study may prove key in understanding the mechanisms involved in the developmental origins of health and disease and identifying novel avenues for prevention and treatment.

As identified in mammalian studies [25, 26], and in the muscle specific grb10 KO in mice, muscle mass is elevated [115]. The *grb10a* zebrafish KD was associated with a lower condition factor than in the SC, which is consistent with greater lean mass. Assessment of skeletal muscle fibre thickness showed only a marginal increase. Therefore, the KD at 18 months has not had a marked impact on this aspect of the phenotype. More detailed studies on body composition would help to clarify the difference in condition factor. However, this study has demonstrated long-term cardiovascular and metabolic changes. The establishment of an altered cardiac phenotype during embryogenesis was followed by a notable change in ventricle morphology. Embryonic zebrafish from the KD group showed significantly lower heart rates, which was followed by compacta thickening and in increase in ventricular density in later-life, associated with a greater cross-sectional area of the myocyte bundles. Gene expression analysis of cardiac tissue further supported these differences between KD and SC fish. The greater degree of *myl7* expression in the absence of an increase in *pcna* expression, together with the increase in cardiac muscle fibre cross-sectional area, suggests the presence of cardiac hypertrophy (muscle size) rather than hyperplasia (cell number). However, future studies should perform measurements on individual myocytes to test this suggestion.

Six genes out of 29 expressed in the hypergraph connectivity models of transcriptomic activity in both SC and KD animals were associated with human cardiometabolic health. This finding represented a significant enrichment of genes with a cardiometabolic impact. The observation that the expression of *APOA1* and *APOB* genes, both involved in lipid metabolism and cardiac health, have reduced function in KD fish supports the consensus of a leaner phenotype and an impact on cardiac physiology [116].The *BLK* gene influences fasting insulin levels and beta cell mass in the pancreas [117]. Its pronounced reduction of impact within the KD transcriptome aligns with the increase in fasting blood glucose observed in KD animals. The *BLK* gene has been associated with Maturity Onset Diabetes of the Young (MODY), a condition characterised by early-onset hyperglycaemia [117]. The largest observed change in hypergraph connectivity in transcriptome was with *SERPINH1*. This gene influences collagen synthesis and deposition and has been linked to cardiac fibrosis [118]. The greatest change in higher order interactions in the hypergraph was observed in the *MMP12* and *MMP3* genes both of which have been shown to be involved in cardiac remodelling and lipid metabolism [119–121]. The use of hypergraph analysis including random walk to identify these genes supports their role in molecular cascades as a co-ordinated and connected response is implied. Wider genomic relationships for other human orthologous genes were examined using phenome wide association. This analysis highlighted pathways related to cholesterol metabolism and lung function, implying a relationship with conditions such as hyperlipidaemia, obesity and asthma.

Aerobic scope was also elevated as a result of *grb10a* KD. This greater aerobic scope suggests the potential energy for non-essential activities is elevated in KDs. It would be interesting to assess the performance of KD zebrafish against their SC counterparts in other energy-heavy tasks, such as swimming and courtship. Lastly, glucose homeostasis control was also altered in KD zebrafish. Glucose uptake was elevated during embryogenesis and energy stores were depleted more rapidly. In later life, fasting blood glucose was increased in the KD compared to the SC. This elevation in glucose has been observed in muscle-specific KO of *GRB10* in mice, supporting the hypothesis that *grb10a* has a role in the regulation of glucose [115]. The raised fasting glucose and the marginally higher difference in glucose levels after a glucose load in the KD compared to the SC indicate a degree of insulin resistance in these adult zebrafish. The elevated AKT demonstrated in this work aligns with raised glucose uptake as AKT signalling facilities the translocation of GLUT4 to the cell membrane [122]. We also noted from analysis of the hypergraph model that Phospho-ERK signalling may be differentially involved in the *grb10a* KD model as FOS, a primary regulator of phospho-ERK activity was absent in the hypergraph model but present in SC. FOS signalling has been noted to impact high glucose induced changes related to cardiomyocyte function [123]. There was no difference in sensitivity to exogenous insulin, inferring that the dose of insulin was likely to have overcome the resistance. Overall adult KD zebrafish when compared to SC are larger in size, have cardiac ventricular muscle thickening, greater maximal metabolic rate and aerobic scope and higher fasting glucose. Taken together this indicates that KD zebrafish have a phenotype that has altered their long-term cardiometabolic health.

Compensatory adaptation involving physiological changes in response to early life stressors allows an organism to adapt and survive. It was notable that the human orthologous genes that overlapped between KD and SC hypergraph models exist as a coherent human interactome module implying co-ordinated adaptation of extracellular matrix, protein-lipid complex and collagen pathways. The compensatory growth mechanism identified in this study is in alignment with a mechanism that enhances immediate survival but may trade-off against long-term health, as shown by the co-ordination of human cardiac health GWAS data with our findings. The zebrafish model used is difficult to compare to classic ideas of human low birth weight (LBW) and early programming of the genomic response. However, catch-up growth consequential to LBW is modelled in the *grb10a* zebrafish KD and the results are compatible with early life adaptation and the recognised predisposition to metabolic syndrome, obesity and cardiovascular disease in adults [124].

This study has limitations: growth in the zebrafish has been documented comprehensively for the embryo, early larval, and juvenile phases up to 30 dpf, and again at 18 months. A more detailed growth trajectory between the end of the juvenile phase at 30 dpf and adulthood would provide greater insight into the transition to the longer, heavier but leaner phenotype seen in the adult grb10a KD. Likewise, the transcriptomic organisation has been analysed over 5 to 30 dpf at the time of greatest growth and maturation, but not in adulthood. In addition, transcriptomic analysis was based on bulk sequencing from tissue anterior to the gills. This emphasises tissue independent gene expression [125]. However, tissue specific transcriptomic analysis to provide greater depth of information could be done. Assessment of the activation of signalling molecules related to insulin signalling was carried out in early life, but not in the juveniles or adults. In addition, as grb10 associates with mitochondria [126], evaluation of the effect of KD on mitochondrial function would be interesting. As indicated above, raised fasting glucose in the adults suggested insulin resistance, and more detailed experiments to assess insulin signalling in the tissues and in-vivo responses to a range of insulin doses would help to clarify mechanisms. The cardiac and aerobic assessments, done at 18 months, could be performed throughout the life-course, and could be expanded to provide deeper phenotyping. In future work the assessment myocyte hypertrophy alongside functional strain/speckle tracking analysis and high-resolution echocardiography could be used to provide a stronger link between cardiac structure and function. Our aim however had been to provide a comprehensive description of the altered regulation of the transcriptome in the KD, combined with evaluation of readily measurable phenotypic characteristics.

## Conclusion

This study shows for the first time that early embryonic transient knockdown of *grb10a* expression is sufficient to cause long-term changes in the growth trajectory, metabolic rate, and cardiac physiology of zebrafish. Significant evidence has been presented here to suggest that *grb10a* plays a previously unidentified role in the coordination of these physiological pathways. Furthermore, this study shows that transient knockdown of a single gene during embryogenesis can cause transcriptome wide remodelling. Remodelling established during embryogenesis provides a basis on which the adult phenotype is formed, demonstrating the potentially long-term impacts of early-life events on the later-life phenotype. This provides the first longitudinal *in vivo* support for the DOHaD hypothesis in *Danio rerio* resulting from the transient knockdown of a single gene involved in the control of growth and metabolism. These long-term alterations may have implications for survival, and the model generated in this study, featuring a short developmental window, and rapid generation time, could play a part in future research into the mechanisms underpinning the fetal origins of human health.

## Supplementary Information

Below is the link to the electronic supplementary material.

Supplementary file1 (XLSX 1688 KB)

Supplementary file2 (DOCX 265 KB)

## Data Availability

Transcriptomic data is available from the Gene Expression Omnibus (GSE162474). R code is available online at: https://github.com/terencegarner/GRB10_KD_ZF.
