# Supplementary material for: Transcriptome remodelling and changes in growth and cardiometabolic phenotype result following Grb10a knockdown in the early life of the zebrafish
Source: Cell Mol Life Sci. 2025 Jul 19;82(1):281. doi: 10.1007/s00018-025-05784-9 (PMC12276197; doi:10.1007/s00018-025-05784-9)

***Supplementary Figure 1. Connectivity and entropy of 20-30 dpf associated genes in the SC and KD zebrafish compared with randomly selected gene sets.* 1a.** Connectivity in the experimental data was significantly greater than in the random iterative data, and greater in the KD data than SC, **1b.** Entropy was significantly lower in the experimental data than the random iterative data. Entropy was lower in the KD than SC, though the reduction in entropy between the experimental data and iterated data was greater in the SC than the KD (All tests - Wilcoxon signed rank test, **** p<0.0001).


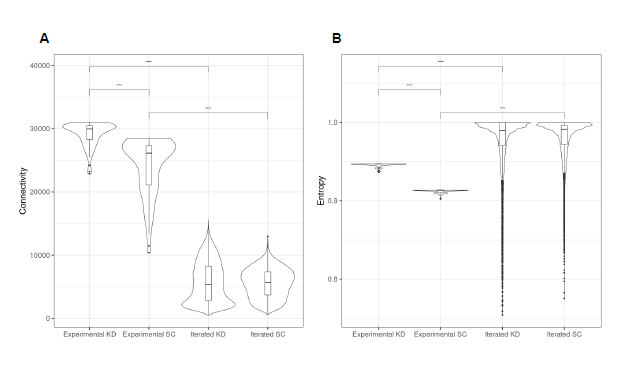


***Supplementary Figure 2. Random walk on hypergraph models of the KD and SC transcriptomes.*** By employing a random walk, transition between nodes in the network can be modelled probabilistically reflecting the flow and coordination present in the transcriptomic system. Random walks were performed on the incidence matrices of the control (SC) and GRB10 KD morpholino hypergraphs independently, restricted to the genes in the central cluster of each as the random walk requires that the hypergraph is not disconnected. Transition matrices were generated for each hypergraph, measuring the probability of a connection between genes, and compared to the hypergraph adjacency matrices produced in previous analyses. The structure of the random walk transition matrix was strongly related to the structure of the hypergraph adjacency matrix, as measured by correlation between the two (control r = 0.63, morpholino r = 0.84)


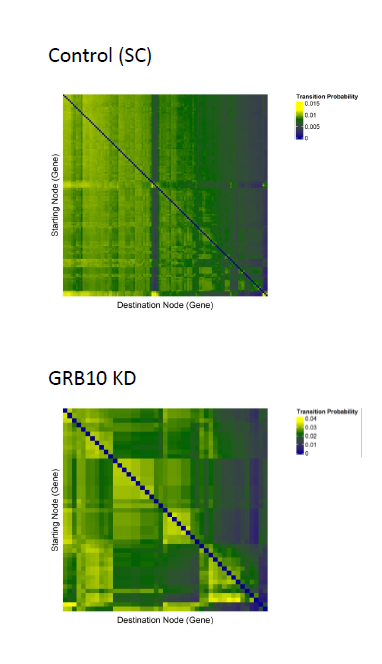


***Supplementary Figure 3. Robustness of hypergraph models for cardiac related human orthologous genes that overlap between KD and SC .*** hypergraph models were permuted 1000 times and the connectivity of the hypergraph (row sum of $M$) was calculated for each distribution and plotted for **A)** SC control and, **B)** KD. The reduced adjacency matrix ($MM^{t}$) was calculated from the incidence matrix iterations (matching) and the size of higher order interactions in the hypergraph was derived from the row sums for **C)** SC control and, **D)** KD. The original hypergraph metrics for cardiac related genes were plotted to the row sum distributions and the z-scores calculated to demonstrate robustness.


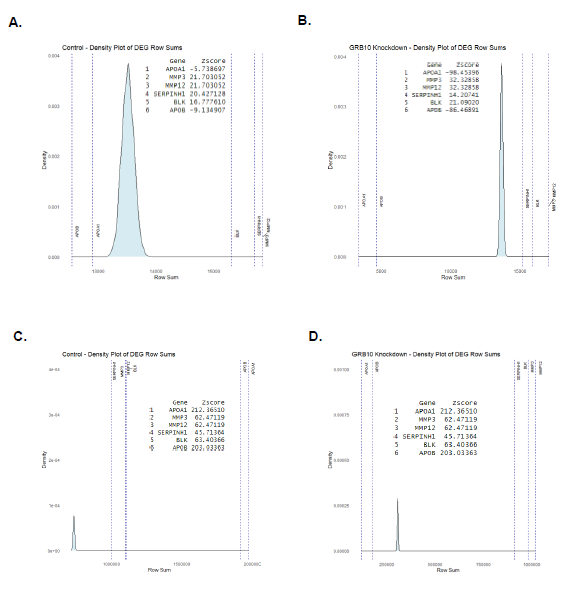

Supplement: Supplementary file 2 — Supplementary file2 (DOCX 265 KB) [file 18_2025_5784_MOESM2_ESM.docx]
